# Post Hoc Analyses of A-SURE and PREVENT Confirm the Effectiveness of rFVIIIFc Prophylaxis Across All Ages, BMIs, Severities, and Inhibitor Histories

**DOI:** 10.1055/a-2894-5837

**Published:** 2026-07-29

**Authors:** Johannes Oldenburg, Andreas Tiede, Jayanthi Alamelu, María Teresa Álvarez Román, Christoph Bidlingmaier, Pål Andre Holme, Flora Peyvandi, Eva Bednar, Eveline Nüesch, Stefan Lethagen

**Affiliations:** 1 Institute of Experimental Haematology and Transfusion MedicineUniversity Hospital Bonn, Medical Faculty, University of BonnBonnGermany; 2 Department of Hematology, Hemostasis, Oncology and Stem Cell TransplantationHannover Medical SchoolHannoverGermany; 3 Paediatric HaematologyEvelina London Children’s Hospital, Guy’s and St Thomas’ NHS Foundation TrustLondonUnited Kingdom of Great Britain and Northern Ireland; 4 Department of HaematologyLa Paz University Hospital-IdiPazMadridSpain; 5 Department of PediatricsDr. von Hauner Children’s Hospital, Pediatric Hemophilia Center, LMU MunichMunichGermany; 6 Department of HaematologyOslo University Hospital, Institute of Clinical Medicine, University of OsloOsloNorway; 7 9304 Fondazione IRCCS Ca’ Granda Ospedale Maggiore Policlinico, Angelo Bianchi Bonomi Haemophilia and Thrombosis CentreMilanItaly; 8 Università degli Studi di Milano, Department of Pathophysiology and Transplantation, and Fondazione Luigi VillaMilanItaly; 9 Global Medical Affairs and Clinical DevelopmentSobiStockholmSweden; 10 BiometricsSobiBaselSwitzerland; 11 Haemostasis and Thrombosis Centre, Copenhagen UniversityCopenhagenDenmark

**Keywords:** hemophilia A, factor VIII, prospective studies

## Abstract

**Objective** To assess the effectiveness of recombinant factor VIII Fc fusion protein (efmoroctocog alfa; referred to herein as rFVIIIFc) prophylaxis in people with hemophilia A (PwHA) stratified by age, body mass index (BMI), disease severity, and inhibitor history included in the A-SURE (NCT02976753) and PREVENT (NCT03055611) real-world studies.

**Methods** PwHA receiving at least one prescription of rFVIIIFc and ≥3 months of follow-up during the prospective periods were included. Post hoc analyses were performed for the overall analysis population and subgroups based on age, BMI, disease severity, and those with and without previous inhibitors. Effectiveness endpoints were annualized bleeding rate (ABR), annualized joint bleeding rate (AjBR), injection frequency, and factor consumption in the prospective period.

**Results** Overall, 333 PwHA were analyzed. ABRs and AjBRs, generally, were low across the different subgroups (range of medians, 0.0–0.6 for both ABRs and AjBRs, except for ABRs in PwHA aged ≥65 years). Injection frequencies ( *n* injections/week) were comparable across subgroups (range of medians, 2.0–2.3), and factor consumption was generally similar (range of medians, 68.3–100.0 international units/kg/week), although with slightly higher factor consumption in pediatric PwHA and lower consumption in the overweight or obese BMI groups.

**Conclusion** These post hoc analyses support the effectiveness and, therefore, use of rFVIIIFc prophylaxis in PwHA across all ages, BMI categories, severities, and for those with a history of inhibitors.

## Introduction

Hemophilia A accounts for approximately 80–85% of all hemophilia cases. 1 People with hemophilia A (PwHA) present with bleeding manifestations as a result of deficiency in factor VIII (FVIII), with the severity of the disease correlated to lower levels of FVIII; severe disease is defined as <1% of normal FVIII levels and nonsevere disease (mild or moderate hemophilia) as FVIII levels of 1–<40%. 1 2 Individuals with hemophilia each present with specific characteristics that require consideration when it comes to treatment management. 1

Treatment considerations can change during a patient’s lifetime, with joint deterioration likely to increase with age, 3 resulting in challenges associated with arthropathy. 4 A higher percentage of PwHA with ≥1 problem joint has been reported in adults when compared with children and adolescents. 3 5 Despite early treatment, bleeds can lead to progressive joint damage. 6 7 A systematic review noted that children and adolescents with hemophilia A tend to have a better quality of life compared with adults, although health-related quality of life (HRQoL) often declines as young individuals grow older. 8 Additionally, people with hemophilia (PwH) may encounter more challenges as they age, including comorbidities such as diabetes and cardiovascular disease, which are reported as more prevalent among older male adults compared with younger adults. 1 9

The prevalence of overweight and obesity among PwH is generally comparable to that of the general population, as observed in Europe and the United States. 10 Over recent decades, rates of overweight and obesity have risen globally, a trend expected to persist. 11 This global rise mirrors the increase observed in both children and adults with hemophilia between 2007 and 2016. 10

Excess body weight can affect joint health both directly, by adding strain to weight-bearing joints, 12 and indirectly, by fostering an inflammatory microenvironment. 13 Joint pain and limited mobility might further contribute to a sedentary lifestyle, complicating weight management. 10 Research findings on the impact of body weight in PwH are mixed. For instance, a single-center study linked higher body mass index (BMI) to poorer ankle joint health, 14 while a post hoc analysis found no significant relationship between BMI and joint health deterioration. 15 When considering bleeding rates, BMI did not appear to significantly influence overall bleeding frequency in one study including PwHA; 16 a trend toward higher annualized bleeding rate (ABR) was observed in individuals of normal weight, compared with those who are overweight or obese. 16 Obese PwHA experienced a significantly higher number of bleeds requiring treatment compared with normal weight controls in another study. 17 This study and one other also showed there was no significant difference in joint bleeds observed between obese PwH and their healthy-weight hemophilia peers. 17 18

People of all severities of hemophilia can experience bleeds, including subclinical bleeds, leading to progressive joint damage and pain. 7 19 20 21 22 23 Although patient-reported outcome measures related to HRQoL are observed to be better in those with mild disease when compared with moderate and severe disease, 20 24 a significant impact on HRQoL is still experienced in those with mild or moderate disease. 22 25 In Nordic countries, there is increased mortality and prevalence of comorbidities, along with higher use of medications for pain, depression, and anxiety, reported across all disease severities, including individuals with mild phenotypes (e.g., males with low factor usage and females, including carriers), compared with matched controls. 25 26

Several treatment options are currently available for the management of hemophilia A, including factor and nonfactor replacement therapies. During FVIII replacement treatment, inhibitors can develop toward administered therapy, 2 which can increase treatment burden and bleeding rates, substantially impacting HRQoL for PwHA, even when they receive usual treatment. 27 28 29 Achieving tolerization to FVIII remains the proposed goal, as, independent of the prophylactic agent, bleeding situations will still require factor replacement; 30 immune tolerance induction is available to help eradicate inhibitors. 1 30

Extended half-life (EHL) FVIII products allow maintenance of sufficiently high FVIII levels to provide protection from bleeds, while also addressing the challenges of prophylactic treatment. 1 Efmoroctocog alfa, a recombinant FVIII Fc fusion protein referred to as rFVIIIFc herein, is an EHL FVIII product that provides higher factor levels, enhancing bleed protection and allowing longer dosing intervals compared with standard half-life (SHL) products. 31 32 33 The efficacy and safety of rFVIIIFc have been thoroughly established through Phase 3 clinical trials involving individuals of all ages with severe hemophilia A. These trials include A-LONG (NCT01181128), 34 Kids A-LONG (NCT01458106), 35 PUPs A-LONG (NCT02234323), 36 and ASPIRE (NCT01454739). 37 However, it is important to note that individuals with nonsevere hemophilia or a history of inhibitors were not included in these clinical studies.

The A-SURE (NCT02976753) and PREVENT (NCT03055611) studies were performed to provide prospective real-world experience as to the effectiveness and usage of rFVIIIFc in clinical practice in Europe, 33 38 resulting in a large and robust pool of data, of which 333 PwHA were analyzed. The A-SURE study utilized a matched control group to compare the effectiveness of rFVIIIFc prophylaxis with SHL FVIII prophylaxis and demonstrated that rFVIIIFc provided improved bleed protection, lower factor consumption, and fewer injections. 33 The PREVENT study demonstrated that rFVIIIFc prophylaxis provides improved bleed protection, with reduced injection frequency and stable factor consumption compared with previous treatment. 38 These studies support the previously presented pharmacokinetic simulation data evaluating prophylaxis with rFVIIIFc compared with a rFVIII. 32

We investigated the real-world effectiveness of rFVIIIFc prophylaxis in subgroups of PwHA with different characteristics, as described earlier, to provide real-world experience relevant to these individual subgroups and to support decision-making in clinical practice. The extensive real-world data collected by the A-SURE and PREVENT studies were utilized to provide data for subgroups that are of clinical relevance but may not always be available in clinical studies. We report the post hoc analyses of a pooled population of PwHA who received rFVIIIFc to assess the effectiveness of rFVIIIFc prophylaxis stratified by age, BMI, disease severity, or inhibitor history.

## Methods

### Study Design

A-SURE (NCT02976753) was a 24-month, prospective, observational study across nine European countries, which aimed to evaluate the real-world effectiveness of prophylaxis with rFVIIIFc in PwHA compared with a matched group receiving prophylaxis with SHL FVIII products, 33 and PREVENT (NCT03055611) was a 24-month prospective, observational study conducted in Germany, which aimed to assess the real-world usage and effectiveness of rFVIIIFc prophylaxis in PwHA. 38 The design of these studies has been previously described, 33 38 with PwHA prescribed rFVIIIFc treatment according to standard clinical practice in both studies.

The objective of the post hoc analyses reported here was to assess the effectiveness of rFVIIIFc prophylaxis in PwHA in a real-world setting stratified by age, BMI, disease severity, or inhibitor history, using final prospective data from the A-SURE and PREVENT studies.

### Population and Outcome Measures

**Fig. 1:**
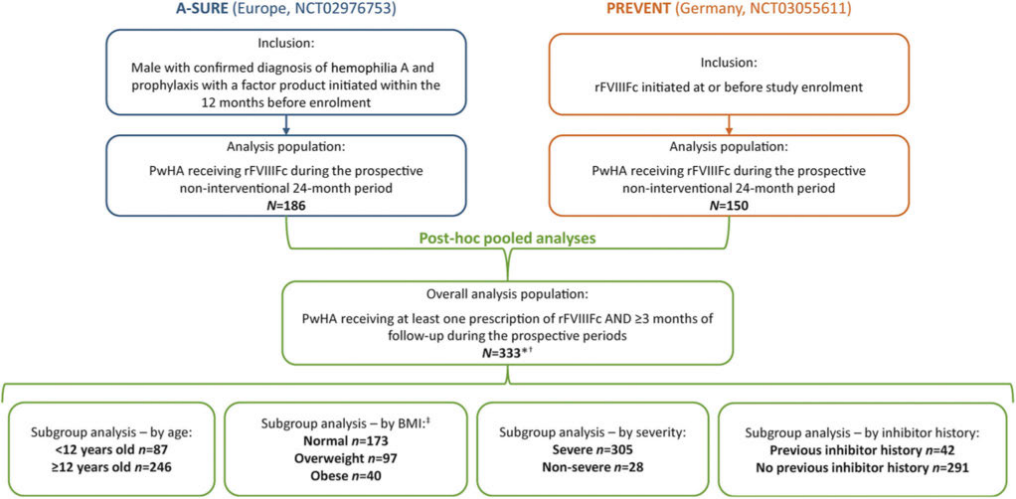
Post hoc analyses design. ^*^ Three PwHA ( *n* = 2, A-SURE; *n* = 1, PREVENT) had <3 months of follow-up during the prospective periods and were excluded from the analyses. ^†^ *n* = 318 PwHA with available BMI data. ^‡^ Due to the small sample size, data from the underweight group were not analyzed further. BMI, body mass index; PwHA, people with hemophilia A; rFVIIIFc, recombinant factor VIII Fc fusion protein.

All PwHA from A-SURE and PREVENT receiving at least one prescription of rFVIIIFc and with ≥3 months follow-up during the prospective periods were pooled and included in the post hoc effectiveness analyses. Analyses were performed in the pooled analysis population, overall and in distinct subpopulations, with subgroups chosen to analyze the effect of age, BMI, disease severity, and inhibitor history on the effectiveness of rFVIIIFc prophylaxis ( Fig. 1 ).

For the overall analysis, population and all subgroups, demographics, baseline characteristics, and length of prospective follow-up were assessed. The effectiveness endpoints of ABR, annualized joint bleeding rate (AjBR), injection frequency ( *n* injections/week), and factor consumption (international unit [IU]/kg/week) in the prospective period were analyzed for the overall analysis population and all subgroups.

For analysis by age, PwHA were grouped into those <12 years of age (pediatric) and those ≥12 years of age (adolescent/adult), with additional analyses performed for the endpoint of ABR across five age subgroups: <6, 6 to 11, 12 to 17, 18 to 64, and ≥65 years. The BMI subgroup analyzed PwHA with available BMI data grouped into predefined categories by baseline BMI ( **Supplementary Table S1** ). PwHA with severe hemophilia (FVIII levels <1%) and nonsevere hemophilia, consisting of those with mild or moderate hemophilia (FVIII levels 1–<40%), were analyzed separately. In the inhibitor history subgroup analysis, previously treated PwHA were grouped into those with a documented history of inhibitors who subsequently tested negative before switching to rFVIIIFc, and those with no prior history of inhibitors. In this subgroup, rFVIIIFc exposure days and inhibitor outcomes were also analyzed.

### Statistical Analyses

Descriptive statistics were used to summarize data. Data analysis was based on available data from the A-SURE and PREVENT studies, and no imputation of missing data was performed.

## Results

### Population Characteristics and Effectiveness—Overall Analysis Population

The overall analysis population included 333 PwHA who received at least one prescription of rFVIIIFc and had ≥3 months of follow-up during the prospective periods in the A-SURE 33 ( *n* = 184) or the PREVENT 38 ( *n* = 149) studies ( Fig. 1 ). Mean (standard deviation [SD]) age was 26.3 (18.3) years, with 26.1% of PwHA <12 years old, 15.0% ≥12 to <18 years old, 55.9% ≥18 to <65 years old, and 3.0% ≥65 years old. Most PwHA had severe hemophilia (305/333, 91.6%), and the most used prior treatment was SHL FVIII (265/269, 98.5%; Table 1 ). The median (interquartile range [IQR]) duration of prospective follow-up for the overall population was 20.9 (19.1–24.0) months, with 21.4 (19.4–24.3) months reported for the A-SURE study analysis population ( *n* = 184) and 20.6 (19.0–22.6) months reported for the PREVENT study analysis population ( *n* = 149).

**Table 1:** Demographic and baseline characteristics of the overall analysis population from A-SURE and PREVENT.

	A-SURE ( *n* = 184)	PREVENT ( *n* = 149)	Overall analysis population ( *n* = 333)
Age ^a^ (y)			
Mean (SD)	27.9 (18.6)	24.4 (17.7)	26.3 (18.3)
Median (min, max)	23.5 (3, 76)	21.0 (0, 74)	22.0 (0, 76)
Age category (y), *n* (%)			
<12	44 (23.9)	43 (28.9)	87 (26.1)
≥12–<18	29 (15.8)	21 (14.1)	50 (15.0)
≥18–<65	103 (56.0)	83 (55.7)	186 (55.9)
≥65	8 (4.3)	2 (1.3)	10 (3.0)
Body weight (kg)	*n* = 182 ^b^	*n* = 143 ^c^	*n* = 325 ^d^
Median (IQR)	69.0 (54.0–81.0)	73.0 (36.0–84.0)	70.0 (47.0–82.0)
BMI category	*n* = 181	*n* = 137	*n* = 318
Underweight	6 (3.3)	2 (1.5)	8 (2.5%)
Normal	104 (57.5)	69 (50.4)	173 (54.4%)
Overweight	45 (24.9)	52 (38.0)	97 (30.5%)
Obese	26 (14.4)	14 (10.2)	40 (12.6%)
Missing	3	12	15
Severity of hemophilia, *n* (%)			
Severe	174 (94.6)	131 (87.9)	305 (91.6)
Moderate	6 (3.3)	17 (11.4)	23 (6.9)
Mild	4 (2.2)	1 (0.7)	5 (1.5)
History of inhibitors, *n* (%)			
Yes	18 (9.8)	24 (16.1)	42 (12.6)
No	166 (90.2)	125 (83.9)	291 (87.4)
Type of prior product, *n* (%)	*n* = 133	*n* = 136	*n* = 269
SHL FVIII	131 (98.5)	134 (98.5)	265 (98.5)
EHL FVIII	2 (1.5)	2 (1.5)	4 (1.5)
Missing	51	13	64

Abbreviations: BMI, body mass index; EHL, extended half-life; FVIII, factor VIII; IQR, interquartile range; SD, standard deviation; SHL, standard half-life.

^a^ Age at enrolment.

^b^ Data missing for *n* = 2.

^c^ Data missing for *n* = 6.

^d^ Data missing for *n* = 8.

Median (IQR) ABR and AjBR of 0.5 (0.0–1.6) and 0.0 (0.0–0.8) were observed, respectively. Median (IQR) injection frequency ( *n* injections/week) was 2.1 (2.0–2.6), with median (IQR) factor consumption of 80.0 (60.6–102.9) IU/kg/week ( **Supplementary Table S2** ).

### Analysis by Age

The analysis included 87/333 (26.1%) PwHA <12 years old and 246/333 (73.9%) PwHA ≥12 years old at enrolment; the age distribution was similar between A-SURE and PREVENT ( Table 1 ). Mean (SD) age was 6.6 (3.4) years and 33.3 (16.2) years in the <12 years and ≥12 years groups, respectively ( **Supplementary Table S3** ). The median (IQR) prospective follow-up for PwHA <12 years ( *n* = 87) was 20.6 (19.1–22.4) months and 21.1 (19.1–24.0) months for those in the ≥12 years group ( *n* = 246).

**Fig. 2:**
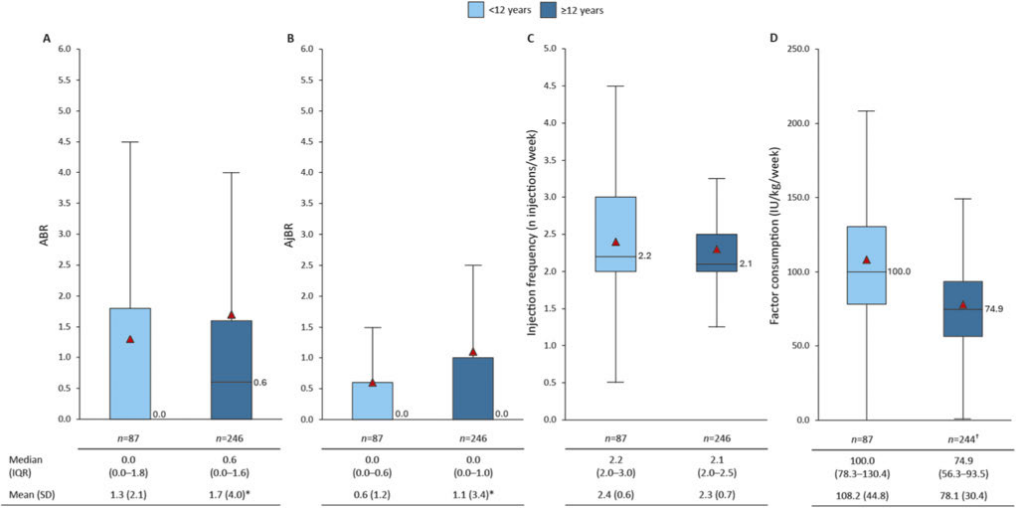
Analyses of ( **A** ) ABR, ( **B** ) AjBR, ( **C** ) injection frequency of rFVIIIFc ( *n* injections/week), and ( **D** ) factor consumption of rFVIIIFc (IU/kg/week) in PwHA aged <12 and ≥12 years. Box plots showing interquartile data range as box boundaries (length of whiskers showing 1.5 times IQR truncated at zero, where applicable), median data (horizontal lines and data labels), and arithmetic means (triangles). ^*^ The sample mean of 246 patients was heavily impacted by one extreme value of 42. ^†^ Data missing for *n* = 2. ABR, annualized bleeding rate; AjBR, annualized joint bleeding rate; IQR, interquartile range; IU, international unit; PwHA, people with hemophilia A; rFVIIIFc, recombinant FVIII Fc fusion protein; SD, standard deviation.

Median ABR and AjBR were comparable between the age groups <12 years and ≥12 years, ranging from 0.0 to 0.6 ( Fig. 2A, B ). When these age groups were split further (at 6, 12, 18 and 65 years), the range of the median ABR remained between 0.0 and 0.6 across all groups except for the ≥65 years subgroup ( **Supplementary Fig. S1** ). Median (IQR) injection frequency ( *n* injections/week) was similar in both age groups ( Fig. 2C ), with the median (IQR) factor consumption (IU/kg/week) for PwHA <12 years slightly higher, compared with PwHA ≥12 years ( Fig. 2D ).

### Analysis by BMI

Among the 318 PwHA with available data, 8/318 (2.5%) PwHA were underweight, 173/318 (54.4%) had normal weight, 97/318 (30.5%) were overweight, and 40/318 (12.6%) were obese ( Table 1 ). Mean (SD) age was 19.6 (11.9) years, 22.5 (16.7) years, 33.6 (18.6) years, and 29.2 (19.3) years in those in the underweight, normal, overweight, and obese groups, respectively ( **Supplementary Table S4** ).

The effectiveness analysis included PwHA from the normal BMI group, overweight group, and obese group. Due to the small sample size, data from the underweight group ( *n* = 8) were not analyzed. Of the overall analysis population, 15 PwHA (4.5%) had no BMI data and were excluded from the effectiveness analysis. The median (IQR) prospective follow-up was 21.0 (19.3–24.0) months, 21.0 (19.3–24.4) and 20.0 (18.9–22.6) in those in the normal ( *n* = 173), overweight ( *n* = 97), and obese ( *n* = 40) groups, respectively.

**Fig. 3:**
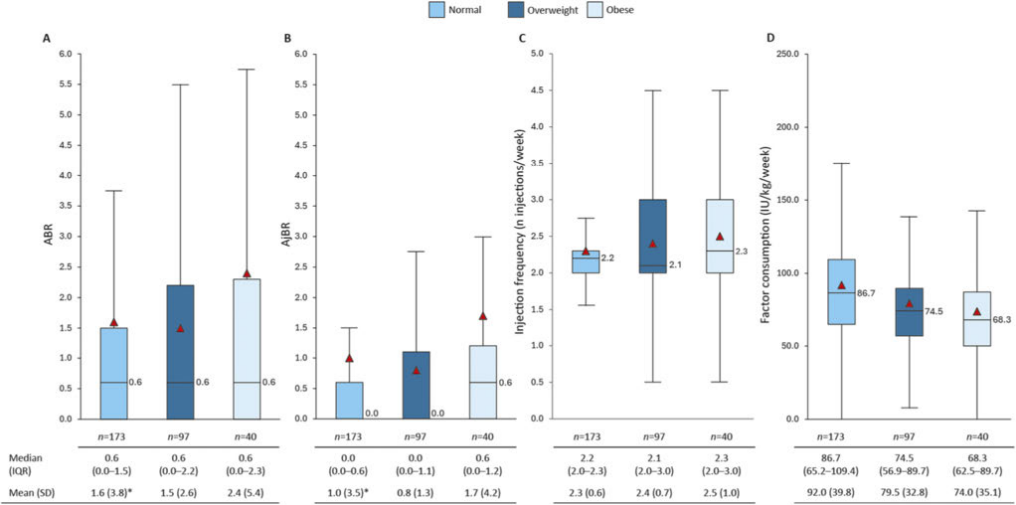
Analyses of ( **A** ) ABR, ( **B** ) AjBR, ( **C** ) injection frequency of rFVIIIFc ( *n* injections/week), and ( **D** ) factor consumption of rFVIIIFc (IU/kg/week) in PwHA by BMI group: normal, overweight, and obese. Box plots showing interquartile data range as box boundaries (length of whiskers showing 1.5 times IQR truncated at zero, where applicable), median data (horizontal lines and data labels), and arithmetic means (triangles). ^*^ The sample mean of 173 patients was heavily impacted by one extreme value of 42. ABR, annualized bleed rate; AjBR, annualized joint bleed rate; BMI, body mass index; IQR, interquartile range; IU, international unit; PwHA, people with hemophilia A; rFVIIIFc, recombinant FVIII Fc fusion protein; SD, standard deviation.

Median (IQR) ABR and AjBR results were generally low across all BMI groups analyzed. However, a higher variability of bleeds and joint bleeds across obese PwHA resulted in slightly higher ABR and AjBR for this group ( Fig. 3A, B ). Median (IQR) injection frequency ( *n* injections/week) was comparable between all three groups ( Fig. 3C ), and median (IQR) factor consumption (IU/kg/week) was lower for the overweight group and obese group, compared with the normal BMI group ( Fig. 3D ).

### Analysis by Hemophilia Severity

Of the 333 PwHA in the severity subgroup, there were 305/333 (91.6%) people with severe hemophilia A and 28/333 (8.4%) with nonsevere hemophilia A; 23 with moderate hemophilia and five with mild hemophilia ( Table 1 ). Mean (SD) age was 26.7 (18.3) years and 22.3 (17.3) years for those with severe hemophilia A and nonsevere hemophilia A, respectively ( **Supplementary Table S5** ). The median (IQR) prospective follow-up for people with severe hemophilia A ( *n* = 305) was 21.0 (19.1–24.0) months, and 20.2 (19.2–22.6) months for those with nonsevere hemophilia A ( *n* = 28).

**Fig. 4:**
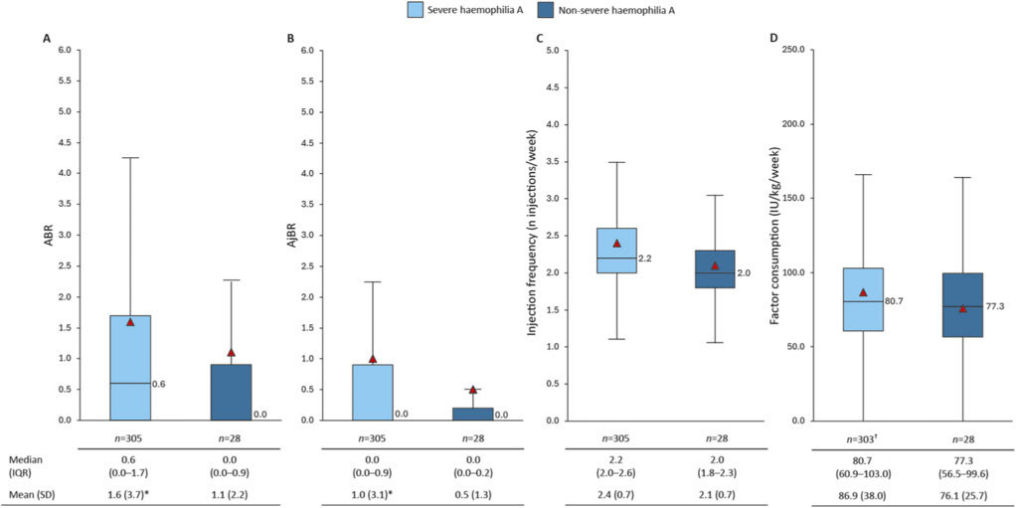
Analyses of ( **A** ) ABR, ( **B** ) AjBR, ( **C** ) injection frequency of rFVIIIFc ( *n* injections/week), and ( **D** ) factor consumption of rFVIIIFc (IU/kg/week) in people with severe and nonsevere hemophilia A. Box plots showing interquartile data range as box boundaries (length of whiskers showing 1.5 times IQR truncated at zero, where applicable), median data (horizontal lines and data labels), and arithmetic means (triangles). ^*^ The sample mean of 305 patients was heavily impacted by one extreme value of 42. ^†^ Data missing for *n* = 2. ABR, annualized bleeding rate; AjBR, annualized joint bleeding rate; IQR, interquartile range; IU, international unit; rFVIIIFc, recombinant FVIII Fc fusion protein; SD, standard deviation.

The range of median ABRs and AjBRs was between 0.0 and 0.6 for both severe and nonsevere hemophilia A, with slightly higher ABR noted for people with severe hemophilia ( Fig. 4A, B ). The median (IQR) injection frequency ( *n* injections/week) and factor consumption (IU/kg/week) were similar in people with severe and nonsevere hemophilia A ( Fig. 4C, D ).

### Analysis by Inhibitor History

The inhibitor history subgroup included 42/333 (12.6%) PwHA with previous inhibitors and 291/333 (87.4%) without previous inhibitors ( Table 1 ). Mean (SD) age was 18.3 (11.1) years and 27.5 (18.8) years in those with previous inhibitors and without previous inhibitors, respectively ( **Supplementary Table S6** ).

The 42 patients with previous inhibitors had more exposure days on rFVIIIFc in total for both retrospective and prospective periods, compared with the 291 patients without ( **Supplementary Table S6** ). The median (IQR) prospective follow-up for PwHA with previous inhibitors ( *n* = 42) was similar to that without previous inhibitors ( *n* = 291), with 21.0 (19.6–23.5) months and 20.9 (19.1–24.0) months of follow-up, respectively.

**Fig. 5:**
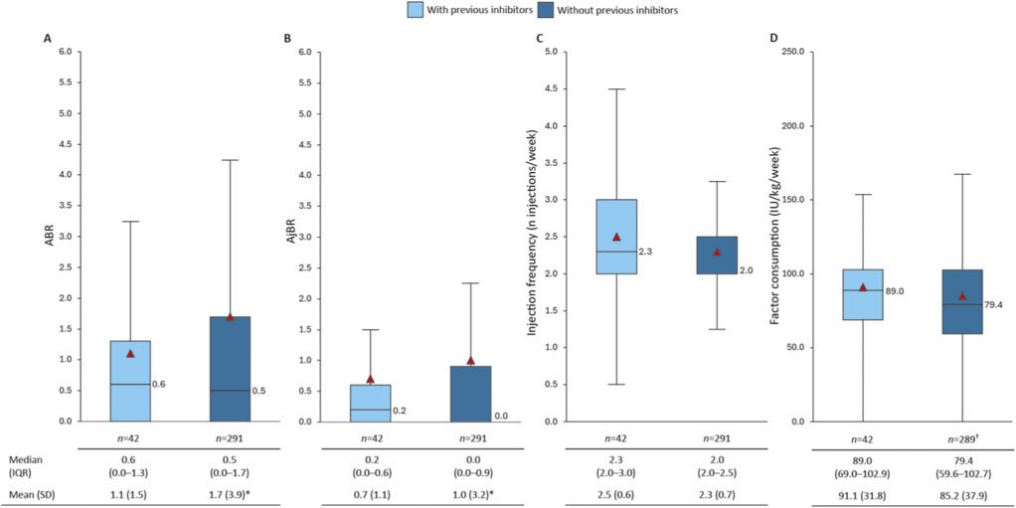
Analyses of ( **A** ) ABR, ( **B** ) AjBR, ( **C** ) injection frequency of rFVIIIFc ( *n* injections/week), and ( **D** ) factor consumption of rFVIIIFc (IU/kg/week) in PwHA with or without previous inhibitors. Box plots showing interquartile data range as box boundaries (length of whiskers showing 1.5 times IQR truncated at zero, where applicable), median data (horizontal lines and data labels), and arithmetic means (triangles). ^*^ The sample mean of 291 patients was heavily impacted by one extreme value of 42. ^†^ Data missing for *n* = 2. ABR, annualized bleed rate; AjBR, annualized joint bleed rate; IQR, interquartile range; IU, international unit; PwHA, people with hemophilia A; rFVIIIFc, recombinant FVIII Fc fusion protein; SD, standard deviation.

Median (IQR) ABR and AjBR for PwHA with previous inhibitors receiving rFVIIIFc were comparable to those without previous inhibitors ( Fig. 5A, B ). The median (IQR) injection frequency ( *n* injections/week) and factor consumption (IU/kg/week) were similar in both groups analyzed ( Fig. 5C, D ). During prospective follow-up on rFVIIIFc prophylaxis, no inhibitors were reported in either the subgroup of PwHA with previous inhibitors (0/42, exact binomial 95% confidence interval [CI]: 0–8.4%) or without previous inhibitors (0/291, exact binomial 95% CI: 0–1.3%).

## Discussion

This study investigated the effectiveness of rFVIIIFc prophylaxis in PwHA in subgroups stratified by age, BMI, disease severity, and presence of previous inhibitors utilizing real-world data from the A-SURE and PREVENT studies. These results provide data that may support clinical decision-making for different PwHA population groups.

rFVIIIFc is approved in PwHA of all ages, 31 and its efficacy and safety have been demonstrated in clinical trials in adolescents/adults and in children. 34 35 36 In the current post hoc analyses on real-world data, results stratified for the different age subgroups highlighted similar ABR and AjBR in those <12 and ≥12 years old. Low ABRs and AjBRs were observed in both adolescent/adult and pediatric PwHA. This is consistent with the interim results reported for the A-MORE study, an ongoing 48-month observational, prospective study evaluating joint health in PwHA receiving rFVIIIFc prophylaxis. 39 40

With regard to factor usage, our analysis reported low injection frequencies and factor consumption in both age subgroups (<12 and ≥12 years). Similar injection frequencies ( *n* injections/week) were observed in both age subgroups in our analysis, with slightly higher factor consumption (IU/kg) among PwHA <12 when compared with those aged ≥12 years. These results are generally consistent with the interim results of the A-MORE study, 39 40 a multicenter observational study from Canada 41 and an Irish real-world experience in pediatric PwHA. 42

It has been acknowledged that there is limited rFVIIIFc data in older PwHA; a post hoc analysis of the A-LONG/ASPIRE clinical studies has been performed and reported that results for the analyzed population of ≥50 years receiving rFVIIIFc prophylaxis were aligned with those from the overall study populations, with low ABR and AjBR. 43 The ABR results for PwHA in the subgroup including adults and older individuals (18–64 years) in the current study were consistent with those for younger PwHA (<18 years), with median ABR of ≤0.6. Although the median (IQR) ABR remained low for all stratified subgroups, a slightly higher ABR was reported for PwHA aged ≥65 years, which may be due to suboptimal treatment earlier in life in this group. These data are based on a small number of PwHA and, therefore, any uncertainty concerning these data should be considered when generalizing these results to other PwHA aged ≥65 years. To prevent bleeding, which may lead to joint damage and disease, primary prophylaxis is recommended immediately or shortly after the first recognized bleed (joint/muscle) in those with severe hemophilia. 44 A database analysis of children with severe hemophilia A reported prophylaxis initiation at a median of 13.1 months in those born between 2010 and 2019, with 62% of children with zero joint bleeds prior to initiation. 45 A delayed start to prophylaxis has been associated with an increased risk of joint damage. 7

The data resulting from the BMI subgroup analysis support the use of rFVIIIFc in PwHA in all BMI categories analyzed. Missing BMI data led to the exclusion of 15 PwHA from the BMI subgroup effectiveness analysis; due to the small sample size ( *n* = 8), data for the underweight BMI group were not analyzed. The slightly higher median ABRs and AjBRs in the obese group, and the observed lower median rFVIIIFc consumption (IU/kg/week) in PwHA in both overweight and obese groups compared with the normal BMI group, may suggest that dosing is not appropriately adjusted in PwHA with a higher BMI.

Median ABRs and AjBRs of ≤0.6 were observed, and both injection frequencies and factor consumptions were similar between the severity subgroups. Observations related to ABR and injection frequency data are consistent with those reported in a systematic literature review on the real-world evidence of rFVIIIFc prophylaxis in PwHA in Europe. 46

Recent literature has highlighted current challenges that exist for people with nonsevere hemophilia, including those relating to disease management and the role of prophylaxis. 47 PwH who have a milder phenotype, PwH with low factor consumption, and women including carriers use more pain, anxiety, and depression medication and have higher comorbidity burden and mortality, compared with the general population. 25 26 It has been suggested that prophylaxis has the potential to improve treatment outcomes with fewer bleeds and the prevention of damage to joints in this population. 48 In our analysis of pooled real-world data, the nonsevere subgroup had a median (IQR) ABR of 0.0 (0.0–0.9) and median (IQR) AjBR of 0.0 (0.0–0.2); this bleed protection combined with the limited burden provided by the reported low injection frequency may suggest that these data support the use of prophylaxis in all PwHA with a predisposed bleeding phenotype independent of their severity status. Physicians have reported switching PwHA with mild, moderate, or severe disease from SHL FVIII to rFVIIIFc, for reasons including fewer injections and to support the individual’s convenience and lifestyle. 49

PwHA with a history of inhibitors have been excluded from previous rFVIIIFc clinical trials (A-LONG, Kids A-LONG and ASPIRE), with no development of inhibitors reported in these study populations of previously treated people with severe hemophilia A. 34 35 37 Inhibitor development was reported in 31.1% of previously untreated people with severe hemophilia A in a Phase 3 trial evaluating the efficacy and safety of rFVIIIFc; this was considered in the expected range for this group. 36 The real-world data presented here demonstrate that the effectiveness of rFVIIIFc was comparable between PwHA with a history of inhibitors and those without previous inhibitors. In addition, PwHA having previously experienced inhibitors were able to tolerate subsequent treatment with rFVIIIFc with no development of inhibitors during the follow-up period. The lack of inhibitor development reported in this study is consistent with findings from 12 real-world experience studies on treatment with rFVIIIFc, of which only one reported low-titer inhibitor development in a previously untreated patient. 46

These post hoc analyses did not aim to address questions around safety, as safety data for rFVIIIFc have been previously reported in the A-SURE and PREVENT primary manuscripts 33 38 and found to be consistent with the established safety profile, including in patients with previous inhibitors, where no inhibitor recurrence was observed.

As the A-SURE and PREVENT studies were conducted in European countries, the study population may not be considered representative of PwHA globally. Moreover, since individuals with nonsevere hemophilia included in the A-SURE and PREVENT studies were required to be receiving regular prophylaxis to participate, this subgroup may not fully represent the broader population of people with mild or moderate hemophilia. However, these PwHA may be representative of those with nonsevere hemophilia, who present with a severe bleeding phenotype. An additional limitation was the exclusion of 15/333 (4.5%) PwHA in the BMI subgroup effectiveness analysis due to missing BMI data; as there was a limited number of excluded patients, no exploration of the impact of missing BMI data was performed. A limited number of PwHA were included in several subcategories, for example, the underweight BMI group ( *n* = 8) or elderly PwHA (aged ≥65 years; *n* = 12). Results of the analysis of PwHA aged ≥65 years are not generalizable to these PwHA subgroups, and further data are needed to confirm the effectiveness of rFVIIIFc in underweight or elderly PwHA.

Generally, low ABRs and AjBRs were observed across the different subgroups investigated. Injection frequencies were comparable across subgroups, and factor consumption was generally similar, except for slightly higher factor consumption observed in PwHA <12 years of age and lower consumption (IU/kg) for those in the overweight or obese BMI groups. The results from the subgroups are in line with those reported for the overall analysis population. These post hoc analyses support the real-world effectiveness and, therefore, use of rFVIIIFc prophylaxis in PwHA across all ages, BMI categories, severities, and for those who have had inhibitors previously, providing additional information on rFVIIIFc effectiveness in population subgroups beyond the overall data available.

## Data Availability

Sobi is committed to responsible and ethical sharing of data on participant level and summary data for medicines and indications approved by the European Medicines Agency and/or U.S. Food and Drug Administration, while protecting individual participant integrity and compliance with applicable legislation. Data access will be granted in response to qualified research requests. All requests are evaluated by a cross-functional panel of experts within Sobi, and a decision on sharing will be based on the scientific merit and feasibility of the research proposal, maintenance of personal integrity, and commitment to publication of the results. To request access to study data, a data sharing request form (available on www.sobi.com) should be sent to medical.info@sobi.com. Further information on Sobi’s data sharing policy and process for requesting access can be found at: https://www.sobi.com/en/policies.

## Supplementary Material

Ergänzendes Material Supplementary Material

## References

[1] Srivastava A Santagostino E Dougall A WFH guidelines for the management of Hemophilia, 3rd edition Haemophilia 2020 26(Suppl 6)1 158 32744769 10.1111/hae.14046

[2] Subcommittee on Factor VIII Blanchette V S Key N S Ljung L R Manco-Johnson M J van den Berg H M Srivastava A Factor IX and Rare Coagulation Disorders of the Scientific and Standardization Committee of the International Society on Thrombosis and Hemostasis. Definitions in hemophilia: communication from the SSC of the ISTH J Thromb Haemost 2014 12 11 1935 1939 25059285 10.1111/jth.12672

[3] Daffunchio C Galatro G Faurlin V Neme D Caviglia H The hidden joint in children with haemophilia on prophylaxis Thromb Res 2023 226 86 92 37130495 10.1016/j.thromres.2023.04.012

[4] Curtis R Manco-Johnson M Konkle B A Comorbidities, health-related quality of life, health-care utilization in older persons with hemophilia-hematology utilization group study part VII (HUGS VII) J Blood Med 2022 13 229 241 35585877 10.2147/JBM.S354526 PMC9109905

[5] McLaughlin P De la Corte-Rodriguez H Burke T An assessment of burden associated with problem joints in children and adults with moderate or severe haemophilia A: analysis of the CHESS-Paediatrics and CHESS II cross-sectional studies Orphanet J Rare Dis 2025 20 01 18 39806420 10.1186/s13023-024-03514-1 PMC11726912

[6] Gringeri A Ewenstein B Reininger A The burden of bleeding in haemophilia: is one bleed too many? Haemophilia 2014 20 04 459 463 24472015 10.1111/hae.12375

[7] Warren B B Thornhill D Stein J Young adult outcomes of childhood prophylaxis for severe hemophilia A: results of the joint outcome continuation study Blood Adv 2020 4 11 2451 2459 32492157 10.1182/bloodadvances.2019001311 PMC7284094

[8] Azeredo-da-Silva A F Zanotto B S Kuwabara Y S Mata V E Quality of life in children and adolescents with hemophilia A: a systematic review and meta-analysis Res Pract Thromb Haemost 2022 7 01 100008 36970745 10.1016/j.rpth.2022.100008 PMC10031335

[9] Soucie J M Le B Dupervil B Poston JN Prevalence of comorbid conditions among older males with haemophilia receiving care in haemophilia treatment centers in the United States Haemophilia 2022 28 06 986 995 35924815 10.1111/hae.14647 PMC10591247

[10] Wilding J Zourikian N Di Minno M Obesity in the global haemophilia population: prevalence, implications and expert opinions for weight management Obes Rev 2018 19 11 1569 1584 30188610 10.1111/obr.12746

[11] GBD 2021 Adult BMI Collaborators Global, regional, and national prevalence of adult overweight and obesity, 1990-2021, with forecasts to 2050: a forecasting study for the Global Burden of Disease Study 2021 Lancet 2025 405(10481)813 838 40049186 10.1016/S0140-6736(25)00355-1 PMC11920007

[12] Chen L Zheng J JY Li G Pathogenesis and clinical management of obesity-related knee osteoarthritis: impact of mechanical loading J Orthop Translat 2020 24 66 75 32695606 10.1016/j.jot.2020.05.001 PMC7349942

[13] Nedunchezhiyan U Varughese I Sun A R Wu X Crawford R Prasadam I Obesity, inflammation, and immune system in osteoarthritis Front Immunol 2022 13 907750 35860250 10.3389/fimmu.2022.907750 PMC9289681

[14] El-Atoum B Prather G Arden D Association between weight status and joint function in adult and pediatric patients with hemophilia Haemophilia 2022 28 04 e101 e104 35279924 10.1111/hae.14541

[15] Kuijlaars I AR Timmer M A de Kleijn P Pisters M F Fischer K Monitoring joint health in haemophilia: factors associated with deterioration Haemophilia 2017 23 06 934 940 28873289 10.1111/hae.13327

[16] Olivieri M Königs C Heller C Prevalence of obesity in young patients with severe haemophilia and its potential impact on factor VIII consumption in Germany Hämostaseologie 2019 39 04 355 359 30722069 10.1055/s-0039-1677874

[17] Biere-Rafi S Haak B W Peters M Gerdes V E Büller H R Kamphuisen P W The impairment in daily life of obese haemophiliacs Haemophilia 2011 17 02 204 208 21332881 10.1111/j.1365-2516.2010.02417.x

[18] Carpenter S L Chrisco M Johnson E The effect of overweight and obesity on joint damage in patients with moderate or severe hemophilia Blood 2006 108 4064

[19] Zwagemaker A F Kloosterman F R Hemke R Joint status of patients with nonsevere hemophilia A J Thromb Haemost 2022 20 05 1126 1137 35171522 10.1111/jth.15676 PMC9314729

[20] Chantrain V A Lambert C De Smet P Pain interferes with daily activities, emotions and sleep in adults with severe, moderate and mild haemophilia: a national cross-sectional survey Haemophilia 2023 29 02 521 529 36657103 10.1111/hae.14747

[21] De la Corte-Rodriguez H Rodriguez-Merchan E C Alvarez-Roman M T Martin-Salces M Rivas-Pollmar I Jimenez-Yuste V Arthropathy in people with mild haemophilia: exploring risk factors Thromb Res 2022 211 19 26 35063801 10.1016/j.thromres.2022.01.010

[22] Chai-Adisaksopha C Noone D Curtis R Non-severe haemophilia: is it benign? – Insights from the PROBE study Haemophilia 2021 27(Suppl 1)17 24 32870546 10.1111/hae.14105

[23] van Leeuwen F HP van Bergen E DP Timmer M A Magnetic resonance imaging evidence for subclinical joint bleeding in a Dutch population of people with severe hemophilia on prophylaxis J Thromb Haemost 2023 21 05 1156 1163 36758725 10.1016/j.jtha.2023.01.035

[24] Ferri Grazzi E Hawes C Camp C Hinds D O’Hara J Burke T Exploring the relationship between condition severity and health-related quality of life in people with haemophilia A across Europe: a multivariable analysis of data from the CHESS II study Health Qual Life Outcomes 2024 22 01 58 39075533 10.1186/s12955-024-02267-6 PMC11288067

[25] Steen Carlsson K Winding B Astermark J High use of pain, depression, and anxiety drugs in hemophilia: more than 3000 people with hemophilia in an 11-year Nordic registry study Res Pract Thromb Haemost 2023 7 02 100061 36908766 10.1016/j.rpth.2023.100061 PMC9999211

[26] Steen Carlsson K Astermark J Baghaei F Comorbidity and mortality in men and women with haemophilia in three Nordic countries-comparisons to matched controls Haemophilia 2025 31 03 401 411 40099856 10.1111/hae.70023 PMC12175107

[27] Mahlangu J Oldenburg J Callaghan M U Health-related quality of life and health status in persons with haemophilia A with inhibitors: a prospective, multicentre, non-interventional study (NIS) Haemophilia 2019 25 03 382 391 31016855 10.1111/hae.13731 PMC6850115

[28] Oldenburg J Shima M Kruse-Jarres R Outcomes in children with hemophilia A with inhibitors: results from a noninterventional study Pediatr Blood Cancer 2020 67 10 e28474 32776489 10.1002/pbc.28474

[29] Mahlangu J Oldenburg J Callaghan M U Bleeding events and safety outcomes in persons with haemophilia A with inhibitors: a prospective, multi-centre, non-interventional study Haemophilia 2018 24 06 921 929 30295389 10.1111/hae.13612

[30] Carcao M Escuriola-Ettingshausen C Santagostino E Future of Immunotolerance Treatment Group. The changing face of immune tolerance induction in haemophilia A with the advent of emicizumab Haemophilia 2019 25 04 676 684 31033112 10.1111/hae.13762 PMC6850066

[31] European Medicines Agency. Elocta. Summary of Product Characteristics, 2025. Accessed February 26, 2025, at:https://www.ema.europa.eu/en/documents/product-information/elocta-epar-product-information_en.pdf

[32] Berntorp E Negrier C Gozzi P Blaas P M Lethagen S Dosing regimens, FVIII levels and estimated haemostatic protection with special focus on rFVIIIFc Haemophilia 2016 22 03 389 396 26863900 10.1111/hae.12887

[33] A-SURE Study Group Oldenburg J Hay C Peyvandi F Superior prophylactic effectiveness of a recombinant FVIIIFc over standard half-life FVIII in hemophilia A: A-SURE study Eur J Haematol 2025 114 02 248 257 39434416 10.1111/ejh.14309 PMC11707817

[34] A-LONG Investigators Mahlangu J Powell J S Ragni M V Phase 3 study of recombinant factor VIII Fc fusion protein in severe hemophilia A Blood 2014 123 03 317 325 24227821 10.1182/blood-2013-10-529974 PMC3894491

[35] Young G Mahlangu J Kulkarni R Recombinant factor VIII Fc fusion protein for the prevention and treatment of bleeding in children with severe hemophilia A J Thromb Haemost 2015 13 06 967 977 25912075 10.1111/jth.12911

[36] Königs C Ozelo M C Dunn A First study of extended half-life rFVIIIFc in previously untreated patients with hemophilia A: PUPs A-LONG final results Blood 2022 139 26 3699 3707 35421219 10.1182/blood.2021013563 PMC9642851

[37] Nolan B Mahlangu J Perry D Long-term safety and efficacy of recombinant factor VIII Fc fusion protein (rFVIIIFc) in subjects with haemophilia A Haemophilia 2016 22 01 72 80 10.1111/hae.12766 26218032

[38] Bidlingmaier C Heller C Langer F Real-world usage and effectiveness of recombinant factor VIII/factor IX Fc in hemophilia A/B: final data from the 24-month, prospective, noninterventional PREVENT study in Germany Res Pract Thromb Haemost 2024 8 05 102482 39101128 10.1016/j.rpth.2024.102482 PMC11295478

[39] Olivieri M Huttunen P Biasoli C Real-world effectiveness and usage of a recombinant factor VIII Fc: interim analysis in children and adolescents from the 48-month perspective, observational A-MORE study Haemophilia 2025 31 72 73

[40] Benitez-Hidalgo O Olsson A Meijer K Real-world effectiveness and usage of a recombinant factor VIII Fc: interim analysis in adults from the 48-month perspective, observational A-MORE study Haemophilia 2025 31 96 97

[41] Sun H L Yang M Poon M C Factor product utilization and health outcomes in patients with haemophilia A and B on extended half-life concentrates: a Canadian observational study of real-world outcomes Haemophilia 2021 27 05 751 759 34160870 10.1111/hae.14369

[42] Prunty K Brady B Kelly I Kavanagh M Ryan C Nolan B Real world experience of Elocta in paediatric patients with severe haemophilia A in Ireland Haemophilia 2018 24 27

[43] Quon D Jackson S Alvarez-Román M T Long-term clinical outcomes of prophylaxis with an rFVIIIFc or rFIXFc in adults aged ≥50 years with hemophilia A or B Blood Adv 2024 8 18 4751 4755 39042888 10.1182/bloodadvances.2023012462 PMC11414653

[44] Fischer K Collins P W Ozelo M C Srivastava A Young G Blanchette V S When and how to start prophylaxis in boys with severe hemophilia without inhibitors: communication from the SSC of the ISTH J Thromb Haemost 2016 14 05 1105 1109 27186714 10.1111/jth.13298

[45] PedNet study group Ljung R de Kovel M van den Berg H M Primary prophylaxis in children with severe haemophilia A and B-implementation over the last 20 years as illustrated in real-world data in the PedNet cohorts Haemophilia 2023 29 02 498 504 36571801 10.1111/hae.14729

[46] Blatný J Nielsen E M Reitzel S B Real-world evidence on efmoroctocog alfa in patients with haemophilia A: a systematic literature review of treatment experience in Europe Haemophilia 2023 29 04 963 974 37243934 10.1111/hae.14797

[47] Factor Think Tank Dolan G Fijnvandraat K Lenting P J Catarino C Lavin M Nonsevere hemophilia: the need for a renewed focus and improved outcomes Semin Thromb Hemost 2025 51 01 58 67 38733982 10.1055/s-0044-1786358 PMC11750352

[48] Iorio A Königs C Reding M T Prophylaxis use of clotting factor replacement products in people with non-severe haemophilia: a review of the literature Haemophilia 2023 29 01 33 44 36224704 10.1111/hae.14676 PMC10091955

[49] van der Sluijs M Huyghe N Wood C Tawil S A survey of physicians’ treatment switching practice in people on long-term prophylaxis for hemophilia in five European countries Curr Med Res Opin 2022 38 01 65 73 34634979 10.1080/03007995.2021.1991901

