# Supplementary material for: Post Hoc Analyses of A-SURE and PREVENT Confirm the Effectiveness of rFVIIIFc Prophylaxis Across All Ages, BMIs, Severities, and Inhibitor Histories
Source: TH Open. 2026 Jul 29;10:a28945837. doi: 10.1055/a-2894-5837 (PMC13463922; doi:10.1055/a-2894-5837)
Supplement: Supplementary file 1 [file 10-1055-a-2894-5837_29276684.pdf]

Supplementary Table S1. BMI categories for adults (≥18 years) and children/adolescents (<18 years).

For those people with haemophilia A <18 years (children/adolescents), BMI grouping was determined based on number of standard deviations away from World Health Organization growth reference BMI data for the given age.

**BMI category**	**Adults (≥18 years)**	**Children/adolescents (<18 years)**
Underweight	<18.5	<–2 SD
Normal	18.5 to <25	–2 SD to <+1 SD
Overweight	25 to 30	+1 SD to <+2 SD
Obese	≥30	≥+2 SD

Abbreviations: BMI, body mass index; SD, standard deviation. Categories determined by WHO Anthroplus (1), utilising the growth curves provided de Onis et al., 2007 (2) and WHO Multicentre Growth Reference Study Group 2006 (3).

1. World Health Organization. WHO AnthroPlus for personal computers manual: software for assessing growth of the world’s children and adolescents. Geneva, Switzerland: World Health Organization; 2009.

2. de Onis M, Onyango AW, Borghi E, Siyam A, Nishida C, Siekmann J. Development of a WHO growth reference for school-aged children and adolescents. Bull World Health Organ. 2007; 85(9): 660-667.

3. WHO Multicentre Growth Reference Study Group. WHO child growth standards: length/height-for-age, weight-for-age, weight-for-length, weight-for-height and body mass

TH Open | DOI 10.1055/a-2894-5837 | © The Author(s).

index-for-age: methods and development. Geneva, Switzerland: World Health Organization; 2006.

Supplementary Table S2. Analyses of ABR, AjBR, injection frequency of rFVIIIFc (n injections/week) and factor consumption of rFVIIIFc (IU/kg/week) in the overall analysis population.

	**Overall analysis population from A-SURE and PREVENT (*N*=333)**
ABR	
Mean (SD)	1.6 (3.6)
Median (IQR)	0.5 (0.0–1.6)
AjBR	
Mean (SD)	1.0 (3.0)
Median (IQR)	0.0 (0.0–0.8)
Injection frequency (n injections/week)	
Mean (SD)	2.3 (0.7)
Median (IQR)	2.1 (2.0–2.6)
Factor consumption (IU/kg/week)	*n*=331*
Mean (SD)	86.0 (37.2)
Median (IQR)	80.0 (60.6–102.9)

*Data missing for *n*=2.

Abbreviations: ABR, annualised bleeding rate; AjBR, annualised joint bleeding rate; IQR, interquartile range; IU, international unit; SD, standard deviation.

TH Open | DOI 10.1055/a-2894-5837 | © The Author(s).

Supplementary Table S3. Demographic and baseline characteristics of PwHA aged <12 years or ≥12 years from the A-SURE and PREVENT study populations.

	**<12 years of age (*n*=87)**	**≥12 years of age (*n*=246)**
Age* (years)		
Mean (SD)	6.6 (3.4)	33.3 (16.2)
Median (min, max)	7.0 (0, 11)	30.0 (12, 76)
Age category (years), n (%)		
<6	35 (40.9)	–
6–11	52 (59.8)	–
12–17	–	50 (20.3)
18–64	–	186 (75.6)
≥65	–	10 (4.1)
Body weight (kg)	*n*=85[†]	*n*=240[‡]
Median (IQR)	24.0 (18.0–37.0)	78.0 (68.0–85.0)
Severity of haemophilia A, n (%)		
Severe	79 (90.8)	226 (91.9)
Moderate	6 (6.9)	17 (6.9)
Mild	2 (2.3)	3 (1.2)
History of FVIII inhibitors, n (%)		
Yes	14 (16.1)	28 (11.8)
No	73 (83.9)	218 (88.6)
Type of prior product, n (%)	*n*=72	*n*=197

TH Open | DOI 10.1055/a-2894-5837 | © The Author(s).

SHL FVIII	70 (97.2)	195 (99.0)
EHL FVIII	2 (2.8)	2 (1.0)
Missing	15	49

*Age at enrolment; †Data missing for *n*=2; ‡Data missing for *n*=6.

Percentages may not add up to 100% due to rounding.

Abbreviations: EHL, extended half-life; FVIII, factor VIII; IQR, interquartile range; PwHA, people with haemophilia A; SD, standard deviation; SHL, standard half-life.

Supplementary Table S4. Demographic and baseline characteristics of PwHA by BMI category from the A-SURE and PREVENT study populations.

	**Underweight (*n*=8)**	**Normal (*n*=173)**	**Overweight (*n*=97)**	**Obese (*n*=40)**
Age* (years)				
Mean (SD)	19.6 (11.9)	22.5 (16.7)	33.6 (18.6)	29.2 (19.3)
Median (min, max)	20.5 (6, 40)	18.0 (0, 76)	31.0 (1, 74)	27.5 (3, 72)
Age category (years), n (%)				
<12	3 (37.5)	55 (31.8)	13 (13.4)	10 (25.0)
≥12–<18	0	31 (17.9)	10 (10.3)	6 (15.0)
≥18–<65	5 (62.5)	85 (49.1)	69 (71.1)	22 (55.0)
≥65	0	2 (1.2)	5 (5.2)	2 (5.0)
Body weight (kg)				
Median	53.0	62.0	74.0	89.0
(IQR)	(20.0–56.0)	(30.0–73.0)	(74.0–89.0)	(68.0–106.5)
Severity of haemophilia A, n (%)				

TH Open | DOI 10.1055/a-2894-5837 | © The Author(s).

Severe	7 (87.5)	158 (91.3)	89 (91.8)	38 (95.0)
Moderate	1 (12.5)	10 (5.8)	8 (8.2)	2 (5.0)
Mild	0	5 (2.9)	0	0
History of FVIII inhibitors, n (%)				
Yes	1 (12.5)	24 (13.9)	8 (8.2)	7 (17.5)
No	7 (87.5)	149 (86.1)	89 (91.8)	33 (82.5)
Type of prior product, n (%)	*n*=7	*n*=135	*n*=80	*n*=35
SHL FVIII	6 (85.7)	133 (98.5)	79 (98.8)	35 (100.0)
EHL FVIII	1 (14.3)	2 (1.5)	1 (1.3)	0
Missing	1	38	17	5

*Age at enrolment.

Percentages may not add up to 100% due to rounding.

Abbreviations: BMI, body mass index; EHL, extended half-life; FVIII, factor VIII; IQR, interquartile range; PwHA, people with haemophilia A; SD, standard deviation; SHL, standard half-life.

Supplementary Table S5. Demographic and baseline characteristics of people with severe or non-severe haemophilia A from the A-SURE and PREVENT study populations.

	**Severe haemophilia A (*n*=305)**	**Non-severe haemophilia A (*n*=28)**
Age* (years)		
Mean (SD)	26.7 (18.3)	22.3 (17.3)

TH Open | DOI 10.1055/a-2894-5837 | © The Author(s).

Median (min, max)	23.0 (0, 76)	14.5 (0, 74)
Age category (years), n (%)		
<12	79 (25.9)	8 (28.6)
≥12–<18	42 (13.8)	8 (28.6)
≥18–<65	175 (57.4)	11 (39.3)
≥65	9 (3.0)	1 (3.6)
Body weight (kg)	*n*=298[†]	*n*=27[‡]
Median (IQR)	70.0 (51.0–82.0)	59.0 (34.0–82.0)
Severity of haemophilia A, n (%)		
Severe	305 (100)	–
Moderate	–	23 (82.1)
Mild	–	5 (17.9)
History of FVIII inhibitors, n (%)		
Yes	41 (13.4)	1 (3.6)
No	264 (86.6)	27 (96.4)
Type of prior product, n (%)	*n*=247	*n*=22
SHL FVIII	243 (98.4)	22 (100.0)
EHL FVIII	4 (1.6)	–
Missing	58	6

*Age at enrolment; [†]Data missing for *n*=7; [‡]Data missing for *n*=1.

Percentages may not add up to 100% due to rounding.

Abbreviations: EHL, extended half-life; FVIII, factor VIII; IQR, interquartile range; SD, standard deviation; SHL, standard half-life.

TH Open | DOI 10.1055/a-2894-5837 | © The Author(s).

Supplementary Table S6. Demographic, baseline characteristic and rFVIIIFc exposure of PwHA by inhibitor history from the A-SURE and PREVENT study populations.

	**With previous inhibitors (*n*=42)**	**Without previous inhibitors (*n*=291)**
Age* (years)		
Mean (SD)	18.3 (11.1)	27.5 (18.8)
Median (min, max)	15.0 (4, 55)	24.0 (0, 76)
Age category at rFVIIIFc initiation (years), n (%)		
<12	14 (33.3)	73 (25.1)
≥12–<18	11 (26.2)	39 (13.4)
≥18–<65	17 (40.5)	169 (58.1)
≥65	–	10 (3.4)
Body weight (kg)		*n*=283†
Median (IQR)	66.0 (37.0–80.0)	71.0 (48.0–82.0)
Severity of haemophilia, n (%)		
Severe	41 (97.6)	264 (90.7)
Moderate	1 (2.4)	22 (7.6)
Mild	–	5 (1.7)
History of FVIII inhibitors, n (%)		
Yes	42 (100.0)	–
No	–	291 (100.0)
Type of prior product, n (%)	*n*=36	*n*=233

TH Open | DOI 10.1055/a-2894-5837 | © The Author(s).

SHL FVIII	36 (100.0)	229 (98.3)
EHL FVIII	–	4 (1.7)
Missing	6	58
EDs on rFVIIIFc		
Mean (SD)	306.1 (94.5)	276.3 (100.2)
Median (IQR)	298.2 (251.7–348.6)	261.5 (211.2–325.0)
Min, max	102–533	35–740

*Age at enrolment; †Data missing for *n*=8.

Percentages may not add up to 100% due to rounding.

ED, exposure day; IQR, interquartile range; PwHA, people with haemophilia A; rFVIIIFc, recombinant factor VIII Fc fusion protein; SD, standard deviation.

TH Open | DOI 10.1055/a-2894-5837 | © The Author(s).

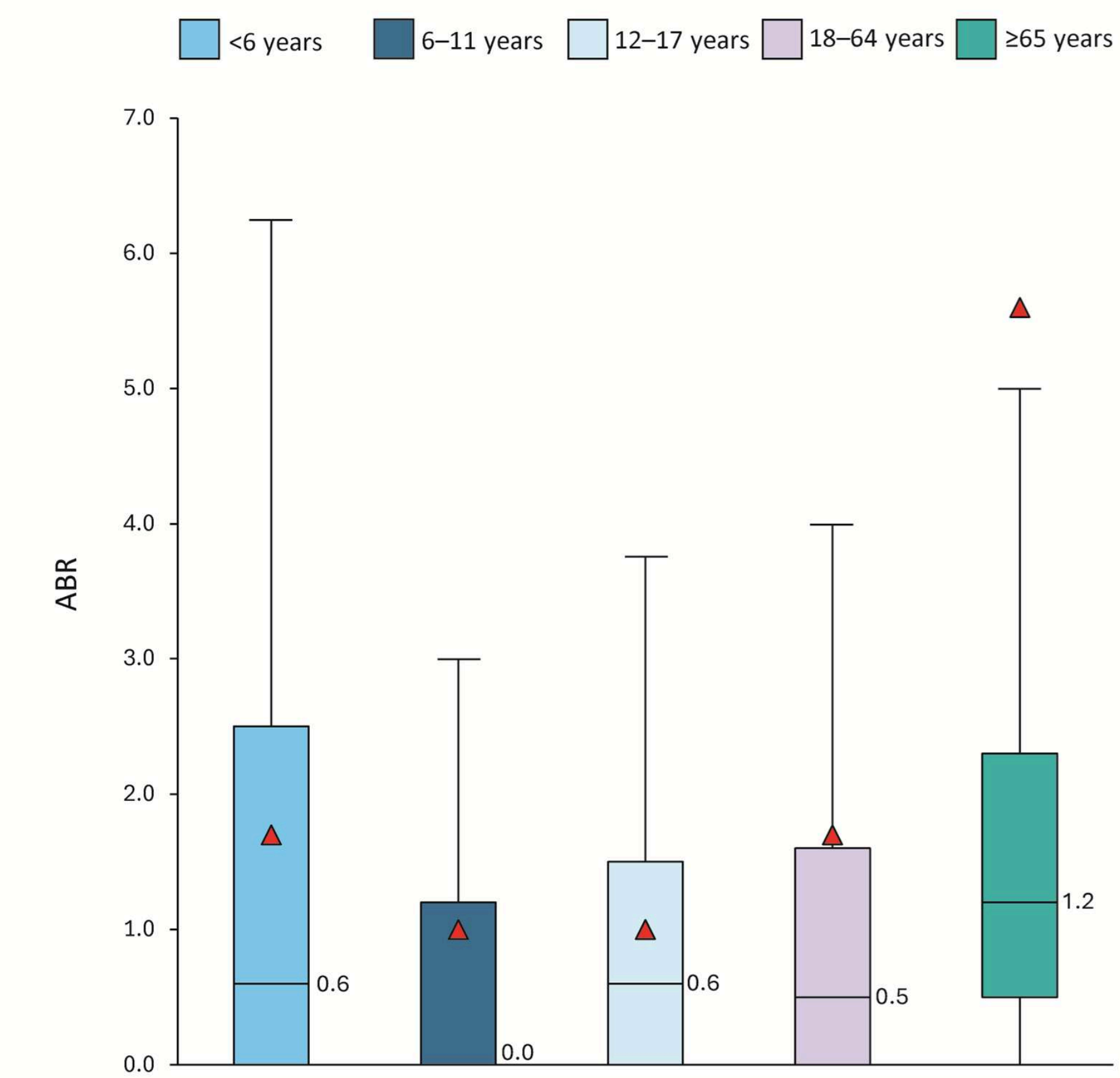

	n=35	*n*=52	*n*=50	*n*=186	*n*=10
Median (IQR)	0.6 (0.0–2.5)	0.0 (0.0–1.2)	0.6 (0.0–1.5)	0.5 (0.0–1.6)	1.2 (0.5–2.3)
Mean (SD)	1.7 (2.5)	1.0 (1.8)	1.0 (1.4)	1.7 (3.5)	5.6 (12.8)*

Supplementary Fig. S1. Analysis of ABR in PwHA aged <6 years, 6–11 years, 12–17 years, 18–64 years and ≥65 years. Box plots showing interquartile data range as box boundaries (length of whiskers showing 1.5 x IQR truncated at zero, where applicable), median data (horizontal lines and data labels), and arithmetic means (triangles). *The sample mean of 10 patients was heavily impacted by one extreme value at 42. Abbreviations: ABR, annualised bleeding rate; IQR, interquartile range; PwHA, people with haemophilia A; SD, standard deviation.
